# Supplementary material for: HIV and Substance Use in Latin America: A Scoping Review
Source: Int J Environ Res Public Health. 2022 Jun 12;19(12):7198. doi: 10.3390/ijerph19127198 (PMC9222977; doi:10.3390/ijerph19127198)
Supplement: Supplementary file 1 [file ijerph-19-07198-s001.zip › ijerph-1727411-supplementary.pdf]

Supplementary S1: Search strategy

Librarians searched in databases from their dates of inception through the last search date of February 4, 2022. The following databases were searched using English language search terms: Global Health (EBSCOhost), Latin American & Caribbean Health Sciences Literature (LILACS), PubMed, and Scopus. Portuguese search terms were used to search in GreenFILE, Health Business FullTEXT, MedicLatina, MEDLINE Complete, Psychology and Behavioral Sciences Collection (EBSCO*host*); LA Referencia; Portal Regional de la BVS; and Scopus. Spanish search terms were used to search in GreenFILE, Health Business FullTEXT, MedicLatina, MEDLINE Complete, Psychology and Behavioral Sciences Collection (EBSCO*host*); LA Referencia; Portal Regional de la BVS; and ProQuest Central.

Search Strategy Report:
Date: 2/4/2022

English Language Searches

Database: PubMed (MEDLINE)

Set #	
1	"Substance-Related Disorders"[Mesh] OR "Alcohol Drinking"[Mesh] OR "Drug Users"[Mesh] OR "Drug Misuse"[Mesh] OR "substance-related disorder"[tiab] OR "substance-related disorders"[tiab] OR "drug use disorder"[tiab] OR "drug use disorders"[tiab] OR "substance use"[tiab] OR "substance abuse"[tiab] OR "substance dependence"[tiab] OR "substance addiction"[tiab] OR "drug abuse"[tiab] OR "drug abuser"[tiab] OR "drug abusers"[tiab] or "drug dependence"[tiab] OR "drug dependency"[tiab] OR "drug addiction"[tiab] OR "drug addict"[tiab] OR "drug addicts"[tiab] OR "drug habituation"[tiab] OR alcohol[tiab] OR alcoholism[tiab] OR alcoholic[tiab] OR alcoholics[tiab] OR cocaine[tiab] OR marijuana[tiab] OR marijuanas[tiab] OR marihuana[tiab] OR marihuanas[tiab] OR cannabis[tiab] OR narcotic[tiab] OR narcotics[tiab] OR opioid[tiab] OR opioids[tiab] OR opiate[tiab] OR opiates[tiab] OR heroin[tiab] OR methamphetamine[tiab] OR methamphetamines[tiab] OR amphetamine[tiab] OR amphetamines[tiab] OR "illicit drugs"[tiab] OR "illicit drug"[tiab] OR "illegal drugs"[tiab] OR "illegal drug"[tiab] OR "recreational drug"[tiab] OR "recreational drugs"[tiab] OR "street drug"[tiab] OR "street drugs"[tiab] OR "drug misuse"[tiab] OR "drug misuses"[tiab] OR "NMUPD"[tiab] OR "Non-Medical Use of Prescription Drugs"[tiab] OR "Non Medical Use of Prescription Drugs"[tiab]
2	"HIV infections"[mesh] OR HIV[mesh] OR "HIV Long-Term Survivors"[Mesh] OR "Anti-HIV Agents"[Mesh] OR "Acquired Immunodeficiency Syndrome"[mesh] OR HIV[tiab] OR "human immunodeficiency virus"[tiab] OR AIDS[tiab] OR "Acquired Immunodeficiency Syndrome"[tiab]
3	"Latin America"[Mesh] OR "Central America"[Mesh] OR "South America"[Mesh] OR "Latin America"[tiab] OR "Latin American"[tiab] OR "Latin Americans"[tiab] OR "Central America"[tiab] OR "Central American"[tiab] OR "Central Americans"[tiab] OR "South America"[tiab] OR "South American"[tiab] OR "South Americans"[tiab] OR Argentina[tiab] OR Argentine[tiab] OR Argentinian[tiab] OR Argentinians[tiab] OR Bolivia[tiab] OR Bolivian[tiab] OR Bolivians[tiab] OR Brazil[tiab] OR Brazilian[tiab] OR Brazilians[tiab] OR Chile[tiab] OR Chilean[tiab] OR Chileans[tiab] OR Colombia[tiab] OR Colombian[tiab] OR Colombians[tiab] OR "Costa Rica"[tiab] OR "Costa Rican"[tiab] OR "Costa Ricans"[tiab] OR Ecuador[tiab] OR Ecuadorian[tiab] OR Ecuadorians[tiab] OR "El Salvador"[tiab] OR Salvadoran[tiab] OR Salvadorans[tiab] OR Galapagos[tiab] OR Guatemala[tiab] OR Guatemalan[tiab] OR Guatemalans[tiab] OR Honduras[tiab] OR Honduran[tiab] OR Hondurans[tiab] OR Mexico[tiab] OR Mexican[tiab] OR Mexicans[tiab] OR Nicaragua[tiab] OR Nicaraguan[tiab] OR Nicaraguans[tiab] OR Panama[tiab] OR Panamanian[tiab] OR Panamanians[tiab] OR Paraguay[tiab] OR Paraguayan[tiab] OR Paraguayans[tiab] OR Peru[tiab] OR Peruvian[tiab] OR Peruvians[tiab] OR Uruguay[tiab] OR Uruguayan[tiab] OR Uruguayans[tiab] OR Venezuela[tiab] OR Venezuelan[tiab] OR Venezuelans[tiab]
4	#1 AND #2 AND #3

5	#4 AND (2012:3000/12/12[pdat])
6	#5 AND (english[Filter] OR portuguese[Filter] OR spanish[Filter])

Database: Scopus

Set #	
1	TITLE-ABS-KEY ("substance-related disorder" OR "substance-related disorders" OR "drug use disorder" OR "drug use disorders" OR "substance use" OR "substance abuse" OR "substance dependence" OR "substance addiction" OR "drug abuse" OR "drug abuser" OR "drug abusers" OR "drug dependence" OR "drug dependency" OR "drug addiction" OR "drug addict" OR "drug addicts" OR "drug habituation" OR alcohol OR alcoholism OR alcoholic OR alcoholics OR cocaine OR marijuana OR marijuanas OR marihuana OR marihuanas OR cannabis OR narcotic OR narcotics OR opioid OR opioids OR opiate OR opiates OR heroin OR methamphetamine OR methamphetamines OR amphetamine OR amphetamines OR "illicit drugs" OR "illicit drug" OR "illegal drugs" OR "illegal drug" OR "recreational drug" OR "recreational drugs" OR "street drug" OR "street drugs" OR "drug misuse" OR "drug misuses" OR "NMUPD" OR "Non-Medical Use of Prescription Drugs" OR "Non Medical Use of Prescription Drugs")
2	TITLE-ABS-KEY (hiv OR "human immunodeficiency virus" OR aids OR "Acquired Immunodeficiency Syndrome")
3	TITLE-ABS-KEY ("Latin America" OR "Latin American" OR "Latin Americans" OR "Central America" OR "Central American" OR "Central Americans" OR "South America" OR "South American" OR "South Americans" OR argentina OR argentine OR argentinian OR argentinians OR bolivia OR bolivian OR bolivians OR brazil OR brazilian OR brazilians OR chile OR chilean OR chileans OR colombia OR colombian OR colombians OR "Costa Rica" OR "Costa Rican" OR "Costa Ricans" OR ecuador OR ecuadorian OR ecuadorians OR "El Salvador" OR salvadoran OR salvadorans OR galapagos OR guatemala OR guatemalan OR guatemalans OR honduras OR honduran OR hondurans OR mexico OR mexican OR mexicans OR nicaragua OR nicaraguan OR nicaraguans OR panama OR panamanian OR panamanians OR paraguay OR paraguayan OR paraguayans OR peru OR peruvian OR peruvians OR uruguay OR uruguayan OR uruguayans OR venezuela OR venezuelan OR venezuelans)
4	#1 AND #2 AND #3
5	#4 AND (LIMIT-TO (PUBYEAR , 2022) OR LIMIT-TO (PUBYEAR , 2021) OR LIMIT-TO (PUBYEAR , 2020) OR LIMIT-TO (PUBYEAR , 2019) OR LIMIT-TO (PUBYEAR , 2018) OR LIMIT-TO (PUBYEAR , 2017) OR LIMIT-TO (PUBYEAR , 2016) OR LIMIT-TO (PUBYEAR , 2015) OR LIMIT-TO (PUBYEAR , 2014) OR LIMIT-TO (PUBYEAR , 2013) OR LIMIT-TO (PUBYEAR , 2012))
6	#5 AND (LIMIT-TO (LANGUAGE , "English") OR LIMIT-TO (LANGUAGE , "Spanish") OR LIMIT-TO (LANGUAGE , "Portuguese"))

Database: Global Health (EBSCO*host*)

Set #	
1	DE "substance abuse" OR DE "drug abuse" OR DE "glue sniffing" OR DE "solvent sniffing" OR DE "alcoholism" OR DE "drug users" OR DE "injecting drug users" OR TI (“substance-related disorder” OR “substance-related disorders” OR “drug use disorder” OR “drug use disorders” OR "substance use" OR “substance abuse” OR “substance dependence” OR “substance addiction” OR “drug abuse” OR “drug abuser” OR “drug abusers” or “drug dependence” OR “drug dependency” OR “drug addiction” OR “drug addict” OR “drug addicts” OR “drug habituation” OR alcohol OR alcoholism OR alcoholic OR alcoholics OR cocaine OR marijuana OR marijuanas OR marihuana OR marihuanas OR cannabis OR narcotic OR narcotics OR opioid OR opioids OR opiate OR opiates OR heroin OR methamphetamine OR methamphetamines OR amphetamine OR amphetamines OR “illicit drugs” OR “illicit drug” OR “illegal drugs” OR “illegal drug” OR “recreational drug” OR “recreational drugs”

	OR “street drug” OR “street drugs” OR “drug misuse” OR “drug misuses” OR “NMUPD” OR “Non-Medical Use of Prescription Drugs” OR “Non Medical Use of Prescription Drugs”) OR AB (“substance-related disorder” OR “substance-related disorders” OR “drug use disorder” OR “drug use disorders” OR "substance use" OR “substance abuse” OR “substance dependence” OR “substance addiction” OR “drug abuse” OR “drug abuser” OR “drug abusers” or “drug dependence” OR “drug dependency” OR “drug addiction” OR “drug addict” OR “drug addicts” OR “drug habituation” OR alcohol OR alcoholism OR alcoholic OR alcoholics OR cocaine OR marijuana OR marijuanas OR marihuana OR marihuanas OR cannabis OR narcotic OR narcotics OR opioid OR opioids OR opiate OR opiates OR heroin OR methamphetamine OR methamphetamines OR amphetamine OR amphetamines OR “illicit drugs” OR “illicit drug” OR “illegal drugs” OR “illegal drug” OR “recreational drug” OR “recreational drugs” OR “street drug” OR “street drugs” OR “drug misuse” OR “drug misuses” OR “NMUPD” OR “Non-Medical Use of Prescription Drugs” OR “Non Medical Use of Prescription Drugs”)
2	DE "HIV infections" OR DE "HIV-1 infections" OR DE "HIV-2 infections" OR DE "human immunodeficiency viruses" OR DE "Human immunodeficiency virus 1" OR DE "Human immunodeficiency virus 2" OR DE "acquired immune deficiency syndrome" OR TI (HIV OR "human immunodeficiency virus" OR AIDS OR "Acquired Immunodeficiency Syndrome") OR AB (HIV OR "human immunodeficiency virus" OR AIDS OR "Acquired Immunodeficiency Syndrome")
3	DE "Latin America" OR DE "Argentina" OR DE "Bolivia" OR DE "Brazil" OR DE "Chile" OR DE "Colombia" OR DE "Costa Rica" OR DE "Cuba" OR DE "Dominican Republic" OR DE "Ecuador" OR DE "El Salvador" OR DE "Guatemala" OR DE "Honduras" OR DE "Mexico" OR DE "Nicaragua" OR DE "Panama" OR DE "Paraguay" OR DE "Peru" OR DE "Puerto Rico" OR DE "Uruguay" OR DE "Venezuela" OR DE "Central America" OR DE "South America" OR DE "Amazonia" OR TI (“Latin America” OR “Latin American” OR “Latin Americans” OR “Central America” OR “Central American” OR “Central Americans” OR “South America” OR “South American” OR “South Americans” OR Argentina OR Argentine OR Argentinian OR Argentinians OR Bolivia OR Bolivian OR Bolivians OR Brazil OR Brazilian OR Brazilians OR Chile OR Chilean OR Chileans OR Colombia OR Colombian OR Colombians OR “Costa Rica” OR “Costa Rican” OR “Costa Ricans” OR Ecuador OR Ecuadorian OR Ecuadorians OR “El Salvador” OR Salvadoran OR Salvadorans OR Galapagos OR Guatemala OR Guatemalan OR Guatemalans OR Honduras OR Honduran OR Hondurans OR Mexico OR Mexican OR Mexicans OR Nicaragua OR Nicaraguan OR Nicaraguans OR Panama OR Panamanian OR Panamanians OR Paraguay OR Paraguayan OR Paraguayans OR Peru OR Peruvian OR Peruvians OR Uruguay OR Uruguayan OR Uruguayans OR Venezuela OR Venezuelan OR Venezuelans) OR AB (“Latin America” OR “Latin American” OR “Latin Americans” OR “Central America” OR “Central American” OR “Central Americans” OR “South America” OR “South American” OR “South Americans” OR Argentina OR Argentine OR Argentinian OR Argentinians OR Bolivia OR Bolivian OR Bolivians OR Brazil OR Brazilian OR Brazilians OR Chile OR Chilean OR Chileans OR Colombia OR Colombian OR Colombians OR “Costa Rica” OR “Costa Rican” OR “Costa Ricans” OR Ecuador OR Ecuadorian OR Ecuadorians OR “El Salvador” OR Salvadoran OR Salvadorans OR Galapagos OR Guatemala OR Guatemalan OR Guatemalans OR Honduras OR Honduran OR Hondurans OR Mexico OR Mexican OR Mexicans OR Nicaragua OR Nicaraguan OR Nicaraguans OR Panama OR Panamanian OR Panamanians OR Paraguay OR Paraguayan OR Paraguayans OR Peru OR Peruvian OR Peruvians OR Uruguay OR Uruguayan OR Uruguayans OR Venezuela OR Venezuelan OR Venezuelans)
4	#1 AND #2 AND #3
5	#4 AND Limiters - Publication Year: 20120101-20221231
6	#5 AND Narrow by Language: - spanish; Castilian; Narrow by Language: - Portuguese; Narrow by Language: - english

Database: LILACS

Set #	
1	("substance-related disorder" OR "substance-related disorders" OR "drug use disorder" OR "drug use disorders" OR "substance use" OR "substance abuse" OR "substance dependence" OR "substance addiction" OR "drug abuse" OR "drug abuser" OR "drug abusers" OR "drug dependence" OR "drug dependency" OR "drug addiction" OR "drug addict" OR "drug addicts" OR "drug

	habituation" OR alcohol OR alcoholism OR alcoholic OR alcoholics OR cocaine OR marijuana OR marijuanas OR marihuana OR marihuanas OR cannabis OR narcotic OR narcotics OR opioid OR opioids OR opiate OR opiates OR heroin OR methamphetamine OR methamphetamines OR amphetamine OR amphetamines OR "illicit drugs" OR "illicit drug" OR "illegal drugs" OR "illegal drug" OR "recreational drug" OR "recreational drugs" OR "street drug" OR "street drugs" OR "drug misuse" OR "drug misuses" OR "NMUPD" OR "Non-Medical Use of Prescription Drugs" OR "Non Medical Use of Prescription Drugs")
2	(hiv OR "human immunodeficiency virus" OR aids OR "Acquired Immunodeficiency Syndrome")
3	("Latin America" OR "Latin American" OR "Latin Americans" OR "Central America" OR "Central American" OR "Central Americans" OR "South America" OR "South American" OR "South Americans" OR argentina OR argentine OR argentinian OR argentinians OR bolivia OR bolivian OR bolivians OR brazil OR brazilian OR brazilians OR chile OR chilean OR chileans OR colombia OR colombian OR colombians OR "Costa Rica" OR "Costa Rican" OR "Costa Ricans" OR ecuador OR ecuadorian OR ecuadorians OR "El Salvador" OR salvadoran OR salvadorans OR galapagos OR guatemala OR guatemalan OR guatemalans OR honduras OR honduran OR hondurans OR mexico OR mexican OR mexicans OR nicaragua OR nicaraguan OR nicaraguans OR panama OR panamanian OR panamanians OR paraguay OR paraguayan OR paraguayans OR peru OR peruvian OR peruvians OR uruguay OR uruguayan OR uruguayans OR venezuela OR venezuelan OR venezuelans)
4	#1 AND #2 AND #3
5	#4 AND Limit to 2012-2022

Portuguese Language Searches

Database: Scopus

Set #	
1	TITLE-ABS-KEY ("Transtornos Relacionados ao Uso de Substâncias" OR "Abuso Oral de Substâncias" OR "Usuários de Drogas" OR "efeitos dos fármacos" OR "Fármacos Anti-HIV " OR "Drogas Ilícitas" OR "Transtornos Relacionados com Narcóticos" OR "Alcoolismo" OR "Alcoólicos" OR “Bebidas Alcoólicas” OR "Consumo de Bebidas Alcoólicas" OR "Abuso de Maconha" OR "Fumar Maconha" OR "Uso da Maconha" OR "Metanfetamina" OR "Transtornos Relacionados ao Uso de Opioides " OR "Dependência de Ópio" OR Papaver OR Ópio OR Cannabis OR "Dependência de Heroína" OR Heroína OR Cocaína OR "Medicamentos sem Prescrição" OR "Transtornos Relacionados ao Uso de Anfetaminas" OR Anfetamina)
2	TITLE-ABS-KEY (HIV OR "vírus da imunodeficiência humana" OR "Infecções por HIV" OR " Síndrome de Imunodeficiência Adquirida")
3	TITLE-ABS-KEY ("América Latina" OR "América Central" OR "América do Sul" OR "Hispano-Americanos" OR argentin* OR bolivia* OR Brasil* OR Brazil* OR chile* OR Colombia* OR "Costa Rica" OR ecuador* OR "El Salvador" OR guatemal* OR hondur* OR mexic* OR nicaragua* OR panam* OR paraguay* OR peru* OR uruguay* OR venezuela OR venezolan*)
4	#1 AND #2 AND #3
5	#4 AND (LIMIT-TO (PUBYEAR , 2022) OR LIMIT-TO (PUBYEAR , 2021) OR LIMIT-TO (PUBYEAR , 2020) OR LIMIT-TO (PUBYEAR , 2019) OR LIMIT-TO (PUBYEAR , 2018) OR LIMIT-TO (PUBYEAR , 2017) OR LIMIT-TO (PUBYEAR , 2016) OR LIMIT-TO (PUBYEAR , 2015) OR LIMIT-TO (PUBYEAR , 2014) OR LIMIT-TO (PUBYEAR , 2013) OR LIMIT-TO (PUBYEAR , 2012))
6	#5 AND (LIMIT-TO (LANGUAGE , "Portuguese")

Databases: GreenFILE, Health Business FullTEXT, MedicLatina, MEDLINE Complete, Psychology and Behavioral Sciences Collection (EBSCO*host*)

Set #	
1	"Transtornos Relacionados ao Uso de Substâncias" OR "Abuso Oral de Substâncias" OR "Usuários de Drogas" OR "efeitos dos fármacos" OR "Fármacos Anti-HIV " OR "Drogas Ilícitas" OR "Transtornos Relacionados com Narcóticos" OR "Alcoolismo" OR "Alcoólicos" OR “Bebidas Alcoólicas” OR "Consumo de Bebidas Alcoólicas" OR "Abuso de Maconha" OR "Fumar Maconha" OR "Uso da Maconha" OR "Metanfetamina" OR "Transtornos Relacionados ao Uso de Opioides " OR "Dependência de Ópio" OR Papaver OR Ópio OR Cannabis OR "Dependência de Heroína" OR Heroína OR Cocaína OR "Medicamentos sem Prescrição" OR "Transtornos Relacionados ao Uso de Anfetaminas" OR Anfetamina
2	HIV OR "vírus da imunodeficiência humana" OR "Infecções por HIV" OR " Síndrome de Imunodeficiência Adquirida"
3	"América Latina" OR "América Central" OR "América do Sul" OR "Hispano-Americanos" OR argentin* OR bolivia* OR Brasil* OR Brazil* OR chile* OR Colombia* OR "Costa Rica" OR ecuador* OR "El Salvador" OR guatemal* OR hondur* OR mexic* OR nicaragua* OR panam* OR paraguay* OR peru* OR uruguay* OR venezuela OR venezolan*
4	#1 AND #2 AND #3
5	#4 AND (LIMIT-TO (PUBYEAR , 2012 - 2022)
6	#5AND (LIMIT-TO (SOURCE , PUBLICACIONES ACADÉMICAS)
7	#6 AND LIMIT-TO (LANGUAGE , "PORTUGUESE")

Database: LA Referencia

Set #	
1	"Transtornos Relacionados ao Uso de Substâncias" OR "Abuso Oral de Substâncias" OR "Usuários de Drogas" OR "efeitos dos fármacos" OR "Fármacos Anti-HIV" OR "Drogas Ilícitas" OR "Transtornos Relacionados com Narcóticos" OR "Alcoolismo" OR "Alcoólicos" OR “Bebidas Alcoólicas” OR "Consumo de Bebidas Alcoólicas" OR "Abuso de Maconha" OR "Fumar Maconha" OR "Uso da Maconha" OR "Metanfetamina" OR "Transtornos Relacionados ao Uso de Opioides" OR "Dependência de Ópio" OR Papaver OR Ópio OR Cannabis OR "Dependência de Heroína" OR Heroína OR Cocaína OR "Medicamentos sem Prescrição" OR "Transtornos Relacionados ao Uso de Anfetaminas" OR Anfetamina
2	HIV OR "vírus da imunodeficiência humana" OR "Infecções por HIV" OR " Síndrome de Imunodeficiência Adquirida"
3	"América Latina" OR "América Central" OR "América do Sul" OR "Hispano-Americanos" OR argentin* OR bolivia* OR Brasil* OR Brazil* OR chile* OR Colombia* OR "Costa Rica" OR ecuador* OR "El Salvador" OR guatemal* OR hondur* OR mexic* OR nicaragua* OR panam* OR paraguay* OR peru* OR uruguay* OR venezuela OR venezolan*
4	#1 AND #2 AND #3
5	#4 LIMITAR (TIPO DE RECURSO, ARTÍCULO)
6	#5AND (LIMIT-TO (PUBYEAR , 2012 - 2022)
7	#6 AND LIMIT-TO (LANGUAGE , "PORTUGUESE")

Database: Portal Regional de la BVS

Set #	
1	TÍTULO – RESUMEN – ASUNTO: "Transtornos Relacionados ao Uso de Substâncias" OR "Abuso Oral de Substâncias" OR "Usuários de Drogas" OR "efeitos dos fármacos" OR "Fármacos Anti-HIV " OR "Drogas Ilícitas" OR "Transtornos Relacionados com Narcóticos" OR "Alcoolismo" OR "Alcoólicos" OR “Bebidas Alcoólicas” OR "Consumo de Bebidas Alcoólicas" OR "Abuso de Maconha" OR "Fumar Maconha" OR "Uso da Maconha" OR "Metanfetamina" OR "Transtornos Relacionados ao Uso de Opioides " OR "Dependência de Ópio" OR Papaver OR Ópio OR Cannabis OR "Dependência de Heroína" OR Heroína OR Cocaína OR "Medicamentos sem Prescrição" OR "Transtornos Relacionados ao Uso de Anfetaminas" OR Anfetamina
2	TÍTULO – RESUMEN – ASUNTO: HIV OR "vírus da imunodeficiência humana" OR "Infecções por HIV" OR " Síndrome de Imunodeficiência Adquirida"
3	TÍTULO – RESUMEN – ASUNTO: "América Latina" OR "América Central" OR "América do Sul" OR "Hispano-Americanos" OR argentin* OR bolivia* OR Brasil* OR Brazil* OR chile* OR Colombia* OR "Costa Rica" OR ecuador* OR "El Salvador" OR guatemal* OR hondur* OR mexic* OR nicaragua* OR panam* OR paraguay* OR peru* OR uruguay* OR venezuela OR venezolan*
4	#1 AND #2 AND #3
5	#4AND (LIMIT-TO (PUBYEAR , 2012 - 2022)
6	#5 AND (LANGUAGE , "PORTUGUESE")

Spanish Language Searches

Database: Proquest Central

Set #	
1	(Marihuana OR droga* OR Cannabis OR Sustancias OR alcohol* OR Anfetami* OR Heroína OR Cocaína OR “Cocaína Crack” OR Papaver OR Opio* OR Medicamentos sin Prescripción OR "Drogas Ilícitas" OR Narcótico*)
2	(VIH OR SIDA OR "Virus de Inmunodeficiencia Humana" OR HTLV)
3	"América Latina" OR "Hispano-Americanos" OR argentin* OR bolivia* OR Brasil* OR chile* OR Colombia* OR "Costa Rica" OR ecuador* OR "El Salvador" OR guatemal* OR hondur* OR mexic* OR nicaragua* OR panam* OR paraguay* OR peru* OR uruguay* OR venezuela OR venezolan*
4	#1 AND #2 AND #3
5	#4 AND (Limitar (Año , 2012 - 2022)
6	#5 AND (Limitar (Idioma, "Español")

Databases: GreenFILE, Health Business FullTEXT, MedicLatina, MEDLINE Complete, Psychology and Behavioral Sciences Collection (EBSCO*host*)

Set #	
1	(Marihuana OR droga* OR Cannabis OR Sustancias OR alcohol* OR Anfetami* OR Heroína OR Cocaína OR “Cocaína Crack” OR Papaver OR Opio* OR Medicamentos sin Prescripción OR "Drogas Ilícitas" OR Narcótico*)

2	(VIH OR SIDA OR "Virus de Inmunodeficiencia Humana" OR HTLV)
3	"América Latina" OR "Hispano-Americanos" OR argentin* OR bolivia* OR Brasil* OR chile* OR Colombia* OR "Costa Rica" OR ecuador* OR "El Salvador" OR guatemal* OR hondur* OR mexic* OR nicaragua* OR panam* OR paraguay* OR peru* OR uruguay* OR venezuela OR venezolan*
4	#1 AND #2 AND #3
5	#4 AND (LIMIT-TO (PUBYEAR , 2012 - 2022)
6	#5AND (LIMIT-TO (SOURCE , PUBLICACIONES ACADÉMICAS)
7	#6 AND LIMIT-TO (LANGUAGE , "ESPAÑOL")

Database: LA Referencia

Set #	
1	(Marihuana OR droga* OR Cannabis OR Sustancias OR alcohol* OR Anfetami* OR Heroína)
2	(VIH OR SIDA OR "Virus de Inmunodeficiencia Humana" OR HTLV)
3	("América Latina" OR "Hispano-Americanos" OR argentin* OR bolivia* OR Brasil* OR chile* OR Colombia* OR "Costa Rica" OR ecuador* OR "El Salvador" OR guatemal* OR hondur* OR mexic* OR nicaragua* OR panam* OR paraguay* OR peru* OR uruguay* OR venezuela OR venezolan*)
4	#1 AND #2 AND #3
5	#4 (LIMITAR (AÑO , 2012 - 2022)
6	#5 LIMITAR (IDIOMA , "ESPAÑOL")

Database: Portal Regional de la BVS

Set #	
1	TÍTULO – RESUMEN – ASUNTO: (Marihuana OR droga* OR Cannabis OR Sustancias OR alcohol* OR Anfetami* OR Heroína)
2	TÍTULO – RESUMEN – ASUNTO: (VIH OR SIDA OR "Virus de Inmunodeficiencia Humana" OR HTLV)
3	TÍTULO – RESUMEN – ASUNTO: "América Latina" OR "Hispano-Americanos" OR argentin* OR bolivia* OR Brasil* OR chile* OR Colombia* OR "Costa Rica" OR ecuador* OR "El Salvador" OR guatemal* OR hondur* OR mexic* OR nicaragua* OR panam* OR paraguay* OR peru* OR uruguay* OR venezuela OR venezolan*
4	#1 AND #2 AND #3
5	#4 LIMITAR POR (AÑO , 2021) OR (AÑO , 2021) OR (AÑO , 2020) OR (AÑO , 2019) OR (AÑO , 2018) OR (AÑO , 2017) OR (AÑO , 2016) OR (AÑO , 2015) OR (AÑO, 2014) OR (AÑO , 2013) OR (AÑO , 2012)
6	#5 LIMITAR POR (IDIOMA, "ESPAÑOL")
